# Blood-based lipidomic signature of severe obstructive sleep apnoea in Alzheimer’s disease

**DOI:** 10.1186/s13195-022-01102-8

**Published:** 2022-11-03

**Authors:** Farida Dakterzada, Iván D. Benítez, Adriano Targa, Anna Carnes, Montse Pujol, Mariona Jové, Olga Mínguez, Rafi Vaca, Manuel Sánchez-de-la-Torre, Ferran Barbé, Reinald Pamplona, Gerard Piñol-Ripoll

**Affiliations:** 1 Unitat Trastorns Cognitius, Clinical Neuroscience Research, IRBLleida-Santa Maria Lleida University Hospital, Rovira Roure n° 44, 25198 Lleida, Spain; 2 Group of Translational Research in Respiratory Medicine, Hospital Universitari Arnau de Vilanova and Santa Maria, IRBLleida, Lleida, Spain; 3 Center for Biomedical Research in Respiratory Diseases Network (CIBERES), Madrid, Spain; 4 Department of Experimental Medicine, University of Lleida-Biomedical Research Institute of Lleida (UdL-IRBLleida), E25198, Lleida, Spain; 5 Department of Nursing and Physiotherapy, Group of Precision Medicine in Chronic Diseases, University Hospital Arnau de Vilanova and Santa María, IRBLleida, Faculty of Nursing and Physiotherapy, University of Lleida, Lleida, Spain

**Keywords:** Alzheimer’s disease, Biomarker, Diagnosis, Lipidomics, Obstructive sleep apnoea, STOP-Bang questionnaire

## Abstract

**Background:**

Obstructive sleep apnoea (OSA) is the most frequent form of sleep-disordered breathing in patients with Alzheimer’s disease (AD). Available evidence demonstrates that both conditions are independently associated with alterations in lipid metabolism. However, it is unknown whether the expression of lipids is different between AD patients with and without severe OSA. In this context, we examined the plasma lipidome of patients with suspected OSA, aiming to identify potential diagnostic biomarkers and to provide insights into the pathophysiological mechanisms underlying the disease.

**Methods:**

The study included 103 consecutive patients from the memory unit of our institution with a diagnosis of AD. The individuals were subjected to overnight polysomnography (PSG) to diagnose severe OSA (apnoea-hypopnea index ≥30/h), and blood was collected the following morning. Untargeted plasma lipidomic profiling was performed using liquid chromatography coupled with mass spectrometry.

**Results:**

We identified a subset of 44 lipids (mainly phospholipids and glycerolipids) that were expressed differently between patients with AD and severe and nonsevere OSA. Among the lipids in this profile, 30 were significantly correlated with specific PSG measures of OSA severity related to sleep fragmentation and hypoxemia. Machine learning analyses revealed a 4-lipid signature (phosphatidylcholine PC(35:4), cis-8,11,14,17-eicosatetraenoic acid and two oxidized triglycerides (OxTG(58:5) and OxTG(62:12)) that provided an accuracy (95% CI) of 0.78 (0.69–0.86) in the detection of OSA. These same lipids improved the predictive power of the STOP-Bang questionnaire in terms of the area under the curve (AUC) from 0.61 (0.50–0.74) to 0.80 (0.70–0.90).

**Conclusion:**

Our results show a plasma lipidomic fingerprint that allows the identification of patients with AD and severe OSA, allowing the personalized management of these individuals. The findings suggest that oxidative stress and inflammation are potential prominent mechanisms underlying the association between OSA and AD.

**Supplementary Information:**

The online version contains supplementary material available at 10.1186/s13195-022-01102-8.

## Introduction

Alzheimer’s disease (AD) is the main cause of dementia and one of the main causes of disability and death after 75 years of age. Given the increase in life expectancy, its prevalence is expected to increase drastically in the coming years [1]. Currently, there is a lack of drugs that can cure or manage the disease long-term. Therefore, it is important to identify all the factors that we can modify to avoid both the appearance and progression of this disease.

In recent years, different sleep disorders, including their duration and quality, have been shown to be risk factors for the development of AD. Of all known sleep disturbances, the presence of obstructive sleep apnoea (OSA) has been consistently identified as a risk factor for AD in population studies AD [2, 3]. Studies on cognitively healthy elderly subjects with OSA have reported increased amyloid β-42 (Aβ-42) and phosphorylated tau (p-tau) levels as measured in the cerebrospinal fluid (CSF) or on positron emission tomography (PET) [4]. In addition, longitudinal studies have reported an increase in the speed of cerebral amyloid accumulation promoted by OSA [5]. Its prevalence in patients with AD ranges between 45 and 90%, presenting as severe OSA in up to 40% of AD patients [6].

Clinically, although some studies have shown that the presence of OSA advances the age of diagnosis of mild cognitive impairment (MCI) and AD, suggesting that it could accelerate the progression of these diseases in the early stages [7], and that continuous positive airway pressure (CPAP) can improve short-term cognitive performance [8], other longitudinal studies have not observed that the presence of OSA worsens the cognitive evolution of patients with mild–moderate AD [9].

The high prevalence of OSA in patients with AD makes the diagnosis of OSA essential, given that it is also a risk factor for hypertension, diabetes, heart failure, stroke or depression, all of which are risk factors for AD [10, 11]. However, the study of sleep in these patients is complex. Although polysomnography (PSG) is the technique of choice, the need to go to the hospital to sleep one or two nights often limits its usefulness in patients with AD and can generate sleep data that do not correspond to the patient’s usual routine at home [12]. In addition, simple screening questionnaires, including the STOP-Bang questionnaire (SBQ) [13] and the Berlin questionnaire (BQ) [14], have been shown to be insufficient for identifying subjects at risk of OSA in this type of population [15]. Thus, the search for new screening tools to detect OSA in this population remains.

Lipidomics is the science of the large-scale determination of individual lipid species in biological samples and has demonstrated great potential in the search for disease-associated biomarkers. OSA is a pathologic condition that is strongly associated with systemic lipid dyshomeostasis [16]. In addition, OSA can increase lipoxidation, as has been evidenced in both the brain and blood of AD patients [17, 18]. Therefore, the identification of systemic alterations in lipid species using high-performance lipidomic platforms could contribute to finding OSA-associated lipid profiles in AD and increase our understanding of the relationship between these two complex pathological conditions.

Therefore, the aim of our study was (i) to evaluate whether the expression pattern of circulating lipids is different between AD patients with and without severe OSA, which would be of great relevance for the practical and noninvasive screening of OSA among patients with AD; (ii) to investigate whether the severity of OSA is correlated with changes in plasma lipid levels; and (iii) to evaluate the diagnostic performance of lipidomics findings in joint use with classic screening tests such as the SBQ.

## Materials and methods

### Study population

This is an ancillary study from trial NCT02814045 that was conducted in the Cognitive Disorders Unit of the Hospital Universitari Santa Maria (Lleida, Spain) from November 2014 to November 2017 to evaluate the cognitive evolution of AD patients with and without OSA after 1 year of follow-up. The patients were recruited prospectively and consecutively according to the eligibility criteria: (1) males and females above 60 years without specific treatment for dementia at the moment of inclusion and with a new diagnosis of mild or moderate AD (Mini-Mental State Examination (MMSE) score ≥20) according to the National Institute on Aging–Alzheimer’s Association (NIA-AA) criteria [19]; (2) absence of visual or hearing problems that, in the investigator’s judgement, would decrease compliance with the neuropsychological examination; (3) signed informed consent from the patient and the responsible caregiver (and/or if applicable, the legal representative if different from the responsible caregiver); and (4) a knowledgeable and reliable caregiver accompanying the patient to all clinic visits during the study.

The exclusion criteria were as follows: (1) a previous diagnosis of OSA treated with CPAP; (2) severe AD, other types of dementia or patients with mild–moderate AD with current acetylcholinesterase inhibitor treatment or memantine; (3) presence of any previously diagnosed sleep disorder: narcolepsy, severe insomnia or chronic lack of sleep; (4) comorbidities such as cancer, severe depression, severe renal or hepatic insufficiency and severe cardiac or respiratory failure; and (5) the presence of excessive somnolence for unknown reasons. All exclusion criteria are available in the paperwork for NCT02814045.

### Study design

Patients with mild–moderate AD who gave consent to participate in the study underwent a detailed interview regarding personal history, a general clinical examination for associated conditions and comorbidities and anthropometric data collection. At baseline, participants were evaluated by a polysomnographic study, and blood and CSF samples were obtained to determine the APOE genotype and the levels of Aβ42, total tau (t-tau) and p-tau, respectively.

Eligible individuals were selected and classified as severe OSA (apnoea-hypopnea index [AHI] ≥30/h) and nonsevere OSA (AHI <30/h) patients based on the PSG findings. Only those with a complete PSG and available blood samples for determining the plasma lipidome were included in the present study.

All participants underwent cognitive assessment using the MMSE [20]. Seventy patients also underwent a semistructured sleep questionnaire that included the SBQ for the detection of OSA. The SBQ comprises 8 items requiring dichotomous responses related to OSA, snoring, tiredness, observed sleep apnoea, high blood pressure, body mass index (BMI), age, neck circumference and sex. The score ranges from 0 to 8, with the highest scores associated with a high probability of OSA. A cut-off score of ≥ 3 is considered high risk of moderate/severe OSA, and <3 is considered low risk [13].

### Clinical variables

The following variables were collected: age, sex, years of education, unhealthy habits (alcohol consumption and smoking), vascular risk factors (hypertension, diabetes mellitus, dyslipidaemia, stroke and heart diseases) and personal psychiatric history. BMI was calculated as body weight (in kg)/height (in m^2^). Excessive daytime sleepiness was evaluated by the Epworth Sleepiness Scale (ESS) and was defined as a total ESS score > 10 [21].

### Polysomnography (PSG)

PSG was performed according to international guidelines to classify the patients as nonsevere OSA (AHI <30/h) or severe OSA (AHI ≥30/h) patients. The following devices were used: an Embletta® sleep monitor (Embla, Canada), a Sibelmed Exea Series 5 (Sibel SAU, Spain), a Philips Respironics Alice 6 LDx (Philips, USA) and an ApneaLink Resmed (Resmed, Canada).

Apnoea was defined as the absence of airflow for more than 10 s. Hypopnea was defined as a reduction in airflow that lasted more than 10 s leading to arousal or oxygen desaturation (represented by a decrease in oxygen saturation greater than 3%). The AHI was defined as the number of apnoea and hypopnea events per hour during the time spent sleeping. CT90 was defined as the percentage of cumulative sleep time with oxyhaemoglobin saturation (SpO2) <90%. The arousal index was defined as the number of awakening events per hour after sleep onset.

### Genetic analysis

DNA was extracted from buffy coat cells using a Maxwell® RCS blood DNA kit (Promega, USA). APOE genotyping was performed using TaqMan® SNP genotyping assays (C_3038793_20 and C_904973_10) and real-time polymerase chain (PCR) according to the manufacturer’s user guide (Publication No. MAN0009593, revision B.0).

### Cerebrospinal fluid (CSF) biomarkers

All patients underwent lumbar puncture between 8:00 and 10:00 am to avoid variations related to the circadian rhythm. Samples were collected in polypropylene tubes, centrifuged at 2000 × g for 10 min at 4°C and stored at −80°C until use. The levels of CSF Aβ42 (Innotest® β-Amyloid (1-42)), t-tau (Innotest® hTAU Ag) and p-tau (Innotest® Phospho-Tau (181P)) were determined by the enzyme immunoassay method according to the manufacturer’s instructions (Fujirebio Europe, Ghent, Belgium). All samples were measured in duplicate and expressed in pg/ml. Samples were obtained with support from IRBLleida Biobank (B.0000682) and PLATAFORMA BIOBANCOS PT17/0015/0027.

### Lipidomic profiling

The plasma lipidome of patients was determined using untargeted lipidomic analysis. The lipids were extracted based on a previously published and validated method [22]. Lipid extracts were analysed via ultrahigh-performance liquid chromatography (UHPLC) coupled with electrospray ionization quadrupole time of flight (ESI-Q-TOF) tandem mass spectrometry (MS/MS) according to a previously published method [23, 24] using an Agilent 1290 liquid chromatography system (Agilent Technologies, Santa Clara, CA, USA) coupled with a 6520 ESI-Q-TOF mass spectrometer (Agilent Technologies, Santa Clara, CA, USA) was used. Data were acquired in both positive and negative ionization modes.

### Lipidic identification

The differentially expressed features were identified in the Human Metabolome Database (HMDB) [25] according to the exact mass and retention time, while the molecular weight tolerance was adjusted to 30 ppm. Potential identities were confirmed by comparison of the exact mass, retention time and MS/MS spectral fragmentation pattern of the class representative internal standards, when available, with a public database using the LC–MS/MS search module of the HMDB web server, as well as Lipidmatch and MSDIAL software [26].

### Pathway enrichment analysis

The annotated differential lipids were searched against the KEGG library of *H. sapiens*. Pathway enrichment analysis was performed through the MetaboAnalyst web service (http://www.metaboanalyst.cat/) [27]. A hypergeometric test was applied for overrepresentation analysis. *p* values regarding significantly affected pathways were adjusted for the false discovery rate (FDR).

### Statistical analyses

Descriptive statistics were used to summarize the characteristics of the study population. Continuous variables were summarized using the mean (standard deviation) for normally distributed data and the median (25th percentile; 75th percentile) for nonnormally distributed data. The normality of the distributions was assessed by the Shapiro–Wilk test. Categorical data were summarized using frequency (percentage). Clinical and sociodemographic characteristics of the patients were compared between groups separated according to the OSA status (AHI ≥ 30 vs. AHI <30) using the *t* test (or an equivalent nonparametric test) or the chi-squared test depending on whether the variables were quantitative or categorical, respectively. Lipid levels were log-transformed for statistical purposes. Linear models with empirical Bayes statistics were used to evaluate differences in lipid levels between groups [28]. Models for differential expression between groups were adjusted for age, sex and body mass index (BMI). Lipids with a significant difference (*p* value <0.05) between groups and a fold change (FC) higher than 1.25 (or lower than 0.8 for downregulated lipids) were considered differentially expressed. Differential expression between study groups was displayed in volcano plots. Correlations between differentially expressed lipids and PSG parameters were evaluated using Pearson’s correlation coefficient. Furthermore, the variable importance, calculated as the average of 50 runs of random forests, was calculated for each differentially expressed lipid.

A feature selection process based on the random forest algorithm [29] was performed to construct a lipidomic signature that predicted severe OSA. This feature selection process is suitable for high-dimensional data and was applied to the differentially expressed lipids identified and repeated 10 times to account for variability in the selection process. The lipids selected in some executions of the process were included in the candidate set for the final predicted model. The candidates were included as predictors in a logistic model with OSA status as a response. The best model, based on the Akaike information criterion (AIC), included the lipids that composed the final lipidomic signature. The accuracy (95% confidence interval (CI)) of the model was estimated and compared. Receiver operating characteristic (ROC) curves were constructed for the lipidomic signature and the reference questionnaire (STOP-Bang), and the area under the ROC curve (AUC) was used as the global discrimination value measure. *p* values <0.05 were considered to indicate statistical significance. All statistical analyses were performed using R software, version 4.0.2.

## Results

### Characteristics of the sample

A total of 103 consecutive mild–moderate AD patients with clinical data and plasma samples were included in the study. The mean (SD) age of the population was 75.49 (5.62) years; 59 (57.28%) participants were women, and the MMSE score was 23.5 (2.38) points. Arterial hypertension was the most frequent vascular risk factor, present in 60 (58.25%) patients, followed by dyslipidaemia in 47 (45.63%) participants and diabetes in 19 (18.44%) participants. Regarding the sleep parameters, the mean ESS was 5.57 (4.09), and the mean AHI was 29.53 (21.85). Sixty-three patients were considered to have nonsevere OSA (AHI <30), and 40 patients were considered to have severe OSA (AHI≥30). The characteristics of the patients at baseline were similar between the severe OSA and nonsevere OSA groups. The baseline characteristics by OSA status are summarized in Table 1.

**Table 1:** Characteristics of Alzheimer’s disease patients according to their obstructive sleep apnoea (OSA) status. *BMI* body mass index, *AD* Alzheimer’s disease, *AHI* apnoea-hypopnea index per hour, *CSF* cerebrospinal fluid, *APOE Ɛ4* apolipoprotein epsilon 4, *MMSE* Mini-Mental State Examination, *ACE* angiotensin-converting enzyme, *OSA* obstructive sleep apnoea, *SaO2* oxygen saturation, *CT90* time with SaO2 <90%

	All (*n*=103)	Non-severe OSA (AHI<30/h) (*n* = 63)	Severe OSA (AHI≥30/h) (*n* = 40)	*p* value
**Demographic characteristics**				
Age at baseline visit (years), median [IQR]	76.0 [72.0; 80.0]	75.0 [71.5; 79.5]	78.0 [72.8; 80.0]	0.14
Gender (female), *n* (%)	61 (59.2%)	42 (66.7%)	19 (47.5%)	0.085
Education (years), mean (SD)	7.31 (2.68)	7.46 (2.32)	7.08 (3.19)	0.511
BMI (kg/m^2^), mean (SD)	27.7 [25.0; 31.1]	27.6 [24.7; 30.6]	28.1 [26.8; 32.4]	0.081
**Medical disorders**				
Hypertension (yes), *n* (%)	60 (58.3%)	37 (58.7%)	23 (57.5%)	0.999
Diabetes (yes), *n* (%)	19 (18.4%)	13 (20.6%)	6 (15.0%)	0.999
Hypercholesterolaemia (yes), *n* (%)	42 (40.8%)	24 (38.1%)	18 (45.0%)	0.625
Depression (yes), *n* (%)	29 (28.2%)	18 (28.6%)	11 (27.5%)	0.999
Smoker				0.662
Nonsmoker, *n* (%)	82 (79.6%)	48 (76.2%)	34 (85.0%)	
Current, *n* (%)	1 (0.97%)	1 (1.59%)	0 (0.00%)	
Former, *n* (%)	20 (19.4%)	14 (22.2%)	6 (15.0%)	
Family history of AD (yes), *n* (%)	40 (38.8%)	21 (33.3%)	19 (47.5%)	0.219
Use of acetylcholinesterase inhibitors or memantine (yes), *n* (%)	98 (95.1%)	60 (95.2%)	38 (95.0%)	0.999
**Polysomnography parameters**				
AHI (events/h), median [IQR]	23.6 [12.2; 47.7]	15.0 [7.47; 20.3]	52.2 [42.3; 62.0]	<0.001
CT90, %	2.20 [0.31; 9.31]	1.12 [0.18; 4.86]	5.80 [1.07; 14.9]	0.004
Mean SaO2, %	93.0 [92.0; 94.0]	93.0 [92.0; 94.0]	93.0 [92.0; 93.0]	0.269
Minimum SaO2, %	84.0 [79.0; 87.0]	86.0 [82.0; 88.0]	81.5 [78.0; 85.0]	0.003
Arousal index, events/h	37.6 [26.2; 49.5]	29.0 [19.7; 40.6]	46.1 [40.0; 55.8]	<0.001
**Epworth Sleepiness Scale (0–24), median [IQR]**	5.00 [2.50; 8.00]	5.00 [2.00; 8.00]	5.00 [3.00; 8.00]	0.773
**STOP-Bang score**				
**MMSE score**	23.0 [22.0; 25.0]	23.0 [22.0; 25.0]	24.0 [22.0; 25.0]	0.512
**AD biomarkers**				
Aβ42 CSF (pg/ml), median [IQR]	493 [399; 580]	489 [393; 584]	505 [406; 564]	0.679
Total tau CSF (pg/ml), median [IQR]	494 [350; 696]	494 [369; 707]	469 [346; 684]	0.676
Phospho-tau CSF (pg/ml), median [IQR]	81.0 [55.4; 97.5]	80.0 [58.0; 95.0]	81.0 [55.1; 98.0]	0.929
ApoE Ɛ4 (carrier), *n* (%)	55 (53.4%)	32 (50.8%)	23 (57.5%)	0.644
**Medications**				
ACE inhibitors, %	32 (31.1%)	21 (33.3%)	11 (27.5%)	0.707
Beta-blockers, %	16 (15.5%)	7 (11.1%)	9 (22.5%)	0.202
Diuretic agents, %	30 (29.1%)	21 (33.3%)	9 (22.5%)	0.339
Calcium-channel blockers, %	13 (12.6%)	9 (14.3%)	4 (10.0%)	0.738
Lipid-lowering agents, %	43 (41.7%)	24 (38.1%)	19 (47.5%)	0.376
Insulin, %	2 (1.94%)	1 (1.59%)	1 (2.50%)	0.999

### Untargeted lipidomic analysis

**Fig. 1:**
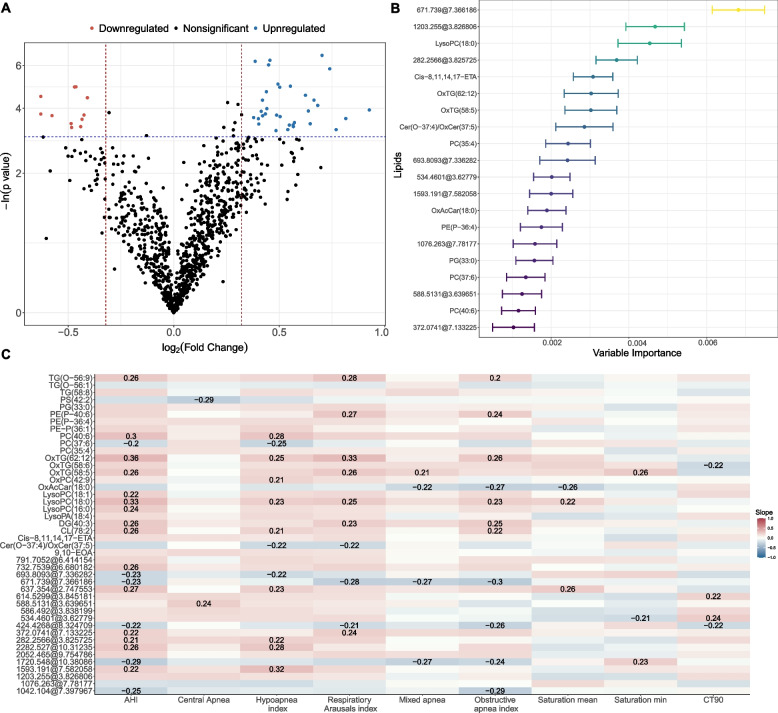
Untargeted lipidomic profiling in AD patients with severe OSA. **A** Volcano plots of the FC (*x*-axis) and *p* value (*y*-axis) for each detected lipid in the comparison of severe OSA vs. nonsevere OSA subjects. Red dots represent significantly downregulated (FC <0.80) molecules, and blue dots represent significantly upregulated (FC> 1.25) molecules in severe OSA patients. The results were adjusted for confounding factors (age, sex and BMI). The *p* value threshold defining statistical significance was <0.05. **B** Top 20 significant lipids in the classification of the study groups (severe OSA vs. nonsevere OSA) based on random forest. **C** Significant correlations between PSG parameters of OSA severity and the differentially expressed lipids. The colour scale illustrates the degree of correlation and ranges from red to blue, indicating negative and positive correlations, respectively. Unknown features are presented as exact mass @ retention time. Definition of abbreviations: AHI apnoea-hypopnea index, LysoPC lysophosphatidylcholine, PG phosphatidylglycerol, FC fold change, OSA obstructive sleep apnoea, PC phosphatidylcholine, PE phosphatidylethanolamine, OxTG oxidized triglyceride, CT90 time with oxygen saturation <90%. For interpretation of the references to colour in this figure legend, the reader is referred to the web version of this article

The first objective of the study was to evaluate the differences in the lipidome in patients with and without severe OSA. Nondirected lipidomics was performed by LC–MS. After quality control, 1022 lipids were detected and included in the analyses. After adjusting for confounding factors (age, sex and BMI), 44 differentially expressed lipid species were identified, 11 with reduced (FC from 0.65 to 0.75) and 33 with increased plasma levels in patients with severe OSA (FC from 1.26 to 1.90) (Fig. 1A and Fig. S1). Subsequently, we identified a lipidomic prediction model for the detection of severe OSA based on random forest analysis. In Fig. 1B, we can see the importance of each lipid in the classification of the study groups (severe OSA vs. nonsevere OSA).

In Fig. 1C, we show the correlation between the different dysregulated lipid species and different parameters of the PSG related to sleep fragmentation (arousal index), different types of apnoea and different measures of hypoxemia (AHI, respiratory arousal index, average and minimum arterial oxygen saturation (SaO2) and CT90. The analysis revealed that of the 44 differentially expressed lipids, 30 were significantly associated with one or more parameters of OSA severity.

### Identification of differentially expressed lipids

Table 2 includes a list of the 25 dysregulated lipid species identified between both groups. These lipid species can be grouped into 14 phospholipids (PLs) (consisting of four phosphatidylcholines (PCs), two lysophosphatidylcholines (lysoPCs), one phosphatidylserine (PS), three phosphatidyl ethanolamine plasmalogens (PE(P)s), one lysophosphatidic acid (lysoPA), one phosphatidic acid (lysoPA) and one cardiolipin (CL)), seven glycerolipids (GLs) (consisting of four triglycerides (TGs), two ether-linked TGs (TG(O)s) and one diglyceride (DG)), 3 fatty acids (consisting of 9,10-epoxyoctadecenoic acid (9,10-EOA), cis-8,11,14,17-eicosatetraenoic acid (8,11,14,17-ETA) and one acylcarnitine) and one sphingolipid (a ceramide). Several lipid species, especially those from the GL category, were oxidized. Nearly all of these oxidized lipids had higher levels in the plasma of AD patients with severe OSA than in nonsevere OSA patients. A list of the all differentially expressed lipid species can be seen in Table S1.

**Table 2:** Putative identity of the significantly differentially expressed lipids between AD patients with and without severe OSA

Putative identity	Class	Mass	RT (min)	Mass/z	Fold change	***p***
LysoPC(18:0)	PL	523.3624	3.63	524.3624	1.628	0.001
PC(40:6)	PL	893.6143	7.40	892.6143	1.307	0.002
DG(40:3)	GL	734.5885	9.16	733.5885	1.365	0.002
OxTG(62:12)	GL	1059.885	9.98	1060.885	1.669	0.003
PE(P-40:6)	PL	775.5565	7.63	776.5565	1.408	0.006
OxPC(42:9)	PL	903.5824	6.56	904.5824	1.467	0.007
Cer(O-37:4)/OxCer(37:5)	SP	587.4894	7.78	588.4894	0.753	0.012
PE(P-36:4)	PL	723.5188	7.25	724.5188	1.259	0.013
OxTG(58:5)	GL	1017.834	9.82	1018.834	1.358	0.019
LysoPC(16:0)	PL	555.3516	2.74	554.3516	1.333	0.021
Cis-8,11,14,17-Eicosatetraenoic acid	FA	364.2588	3.83	363.2588	1.402	0.024
PC(37:6)	PL	791.5414	6.55	792.5414	0.671	0.024
TG(O-56:9)	GL	924.7008	7.11	925.7008	1.339	0.024
PG(33:0)	PL	737.5363	7.51	736.5363	1.418	0.025
PC(35:4)	PL	749.5353	7.33	748.5353	1.32	0.027
TG(O-56:1)	GL	902.8486	7.30	903.8486	0.74	0.028
LysoPC(18:1)	PL	521.3469	2.89	522.3469	1.287	0.03
PS(42:2)	PL	853.6181	7.39	852.6181	0.714	0.032
OxTG(58:6)	GL	1015.822	9.66	1016.822	1.566	0.032
PE(P-36:1)	PL	729.5663	8.11	728.5663	1.48	0.034
LysoPA(18:4)	PL	430.2058	3.62	429.2058	1.481	0.036
OxAcCar(18:0)	FA	443.3681	4.07	444.3681	0.715	0.037
TG(58:8)	GL	990.7796	10.02	989.7796	1.454	0.04
9,10-Epoxyoctadecenoic acid	FA	278.2241	2.62	277.2241	1.705	0.04
CL(78:2)	PL	1605.186	7.56	1604.186	1.334	0.043

Definition of abbreviations: *GL* glycerolipid, *PL* phospholipid, *FA* fatty acid, *SP* sphingolipid, *LysoPC* lysophosphatidylcholine, *PC* phosphatylcholine, *OxPC* oxidized phosphatidylcholine, *DG* diglyceride, *TG* triglyceride, *OxTG* oxidized triglyceride, *PE(P)* phosphatidylethanolamine plasmalogen, *Cer* ceramide, *OxCer* oxidized ceramide, *PG* phosphatidylglycerol, *PS* phosphatidylserine, *LysoPA* lysophosphatidic acid, *OxAcCar* oxidized acylcarnitine, *CL* Cardiolipin

### Lipidomic prediction model for severe OSA

**Fig. 2:**
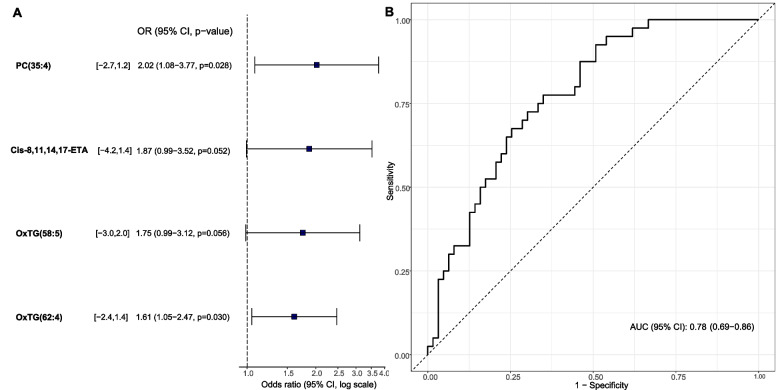
Potential lipid signature for the diagnosis of severe OSA in AD patients. **A** Lipids included in the final model. Feature selection was based on a combination of random forest and automated model selection using the Akaike information criterion. **B** Receiver operating characteristic curves for predicting severe OSA using the lipid signature. The AUC (95% CI) for the model is shown. Definition of abbreviations: PC phosphatidylcholine, Cis-8,11,14,17-ETA Cis-8,11,14,17-eicosatetraenoic acid, OxTG oxidized triglyceride

**Fig. 3:**
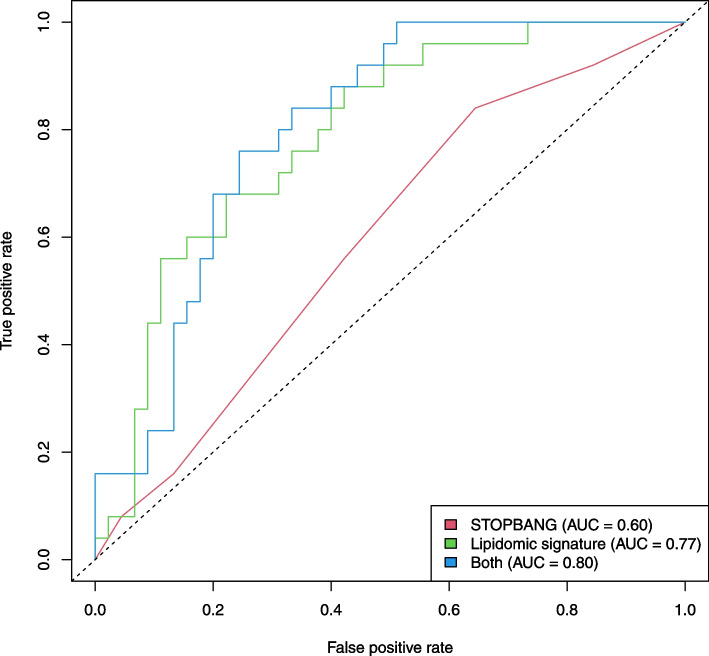
Receiver operating characteristic curves for predicting severe OSA in AD patients using the lipid signature (green), STOP-Bang questionnaire (red) and the combination of both the lipid signature and the STOP-Bang questionnaire (blue). Analysis was performed in those patients with STOP-Bang data. The AUC for each model is shown

The next objective of this study was to identify a plasma lipidomic signature that would allow the identification of subjects with severe OSA. The 25 differentially expressed and identified lipid species were included in the construction of prediction models using machine learning approaches. The multivariate analysis of variable selection based on the random forest revealed a specific fingerprint of severe OSA composed of 4 lipid species (Fig. 2): PC (35:4), 8,11,14,17-ETA and two oxidized TGs (OxTG (58:5) and OxTG (62:12)). The predictive performance of this fingerprint showed an AUC of 0.78 (95% CI 0.69–0.86) in the detection of severe OSA in patients with AD. Addition of the lipid signature data to the information provided by the SBQ (AUC = 0.61 (95% CI 0.50–0.74)) increased the predictive potential for severe OSA (AUC = 0.80 (95% CI 0.70–0.90)) (Fig. 3).

### Integrated analysis of lipid pathways in severe OSA

**Fig. 4:**
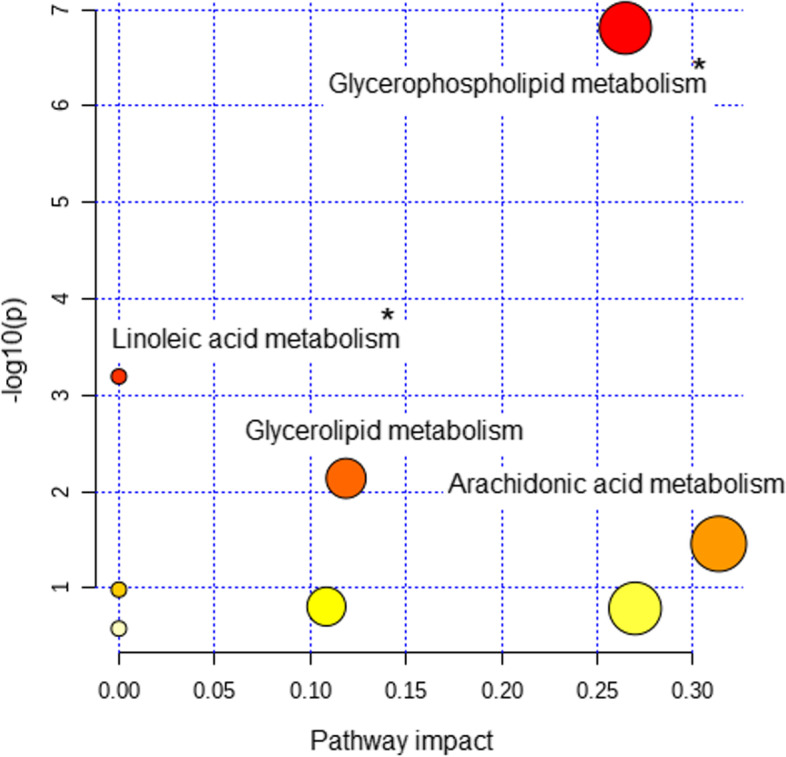
Pathway enrichment analysis of annotated features related to OSA severity in AD patients. Scatter plot presenting the enriched lipid pathways in which classes representative of all identified lipids are involved. Each circle represents a pathway. The colour gradient indicates the significance of the pathway ranked by *p* value, with yellow indicating lower values and red indicating higher values (*y*-axis). The size of the circles represents the impact score of the pathway based on the number of molecules contained in the pathway (*x*-axis). Significantly affected pathways appear with their name. *Significant after FDR correction (*p* <0.05)

Pathway enrichment analysis, using a class representative of all dysregulated lipid features with a putative identity as input, was conducted using the MetaboAnalyst platform. As shown in Fig. 4, this integrated analysis approach yielded four significantly enriched pathways, including PL metabolism, linoleic acid metabolism, GL metabolism and arachidonic acid metabolism. In addition, the *p* values for PL metabolism and linoleic acid metabolism remained significant after FDR correction (*p* = 1.3086E−5 and 0.027, respectively).

## Discussion

In this study, we identified a lipidomic profile associated with the presence of severe OSA in patients with AD. This lipidomic profile was significantly correlated with different polysomnographic measures related to sleep fragmentation and hypoxemia related to OSA severity. We identified 25 plasma lipids that were found in different amounts between AD patients with and without severe OSA, regardless of the incorporation of confounding factors. The differentially expressed lipids were mostly phospholipids, suggesting that cellular membranes are especially susceptible to OSA-mediated pathological alterations. In the machine learning multivariate analyses, the plasma levels of these lipid species allowed us to discriminate those subjects with severe OSA with a much greater capacity than through the use of the STOP-Bang screening questionnaire alone.

AD is a neurodegenerative disease whose appearance and evolution are associated with a number of factors, such as β-amyloid, tau, inflammation, arterial hypertension and diet [30]. In recent years, certain sleep disorders, such as OSA, have been shown to be risk factors that favour the accumulation of amyloid [5] and the appearance of the disease [2, 3]. Up to 40% of patients with mild–moderate AD present with severe OSA; however, the study of this association has not been generalized to memory units in the clinical setting [15].

No published data have shown that the presence of OSA in the dementia phase of AD indicates a worse prognosis at the cognitive level [9]. The underdiagnosis of chronic disorders such as OSA represents an additional cost for health systems compared to the adequate diagnosis and treatment of these diseases [31], and in the specific case of OSA, we know that it is associated with greater difficulties in the control of blood pressure, increased insulin resistance, metabolic syndrome or obesity, all factors that worsen the cognitive evolution of patients [32, 33]. Consequently, and because of the difficulties in performing PSG both due to its economic cost and the relative noncompliance of patients with cognitive impairment, together with the limited usefulness of screening tests [15] in this population, it is justified to explore new peripheral biomarkers that allow the detection of severe forms of OSA.

The brain is mainly composed of lipids, and alterations in lipid composition have been described in AD [34]. OSA has been shown to alter lipid composition at the systemic level and increase lipoxidation [35, 36]. For this reason, we performed an undirected lipidomic analysis with liquid chromatography-tandem mass spectrometry (LC–MS/MS) to robustly and reliably discover biomarkers, as has been done with a great variety of diseases [37, 38]. In our study, we observed how a signature composed of 4 lipid species could serve as a biomarker of severe OSA in patients with AD and be of significantly higher value than the STOP-Bang questionnaire alone, which, to date, has been the most effective screen tool for this population group [15]. These results could allow the identification of those subjects with AD with a high probability of presenting severe OSA and who therefore could be confirmed by PSG. These results would allow the personalized management of patients with AD with high suspicion of severe OSA.

Although lipidomics has been used to study different aspects of AD, especially those related to the diagnosis and differential diagnosis with other types of dementia [39–41], this technique has not been used in the search for biomarkers for the diagnosis of OSA in the AD population. In the population without cognitive impairment, there are also few studies on nondirected metabolomics/lipidomics in blood. Ferrarini et al. [42], in a sample of 33 subjects, identified 14 metabolites, including platelet-activating factor and lysophospholipids, together with some compounds related to the differential activity of the gut microflora (bile pigments and pipecolic acid), that distinguished the severity of OSA; however, they did not analyse the performance of these metabolites as biomarkers of OSA. Lebkuchen et al. [43] identified the differential plasma levels of 22 lipid species in subjects with an AHI> 15 events/hour. Pinilla et al. attempted to overcome some of the limitations of these studies, such as the low reliability due to small sample sizes, the cross-sectional designs and the lack of adjustment for confounding variables [44]. In their study, Pinilla et al. identified a plasma profile composed of 33 metabolites, including lipids, in OSA vs. non-OSA patients. In accordance with our results, in the latter study, PLs were the most affected group of lipids in OSA [45].

To our knowledge, our study is the first attempt to investigate the OSA-associated lipid profile in a population with a high prevalence of this pathological condition, such as patients with AD. Our analysis in an extensive cohort of subjects with mild–moderate AD revealed that despite adjusting for different covariates to eliminate the effect of confounding variables, there were significant alterations in plasma lipid levels due to the presence of severe OSA. In addition, the majority of differentially expressed lipids were also strongly correlated with different PSG measures, especially with variables related to hypoxemia and sleep fragmentation (AHI, mean SaO2, respiratory arousal index, minimum SaO2 and CT90).

The majority of identified lipid species associated with OSA severity were PLs. Pathway enrichment analysis revealed that the metabolism of these lipids was significantly dysregulated. PLs are major constituents of cellular membranes. They provide an optimal environment for the interaction of proteins, their trafficking and their function. The regulation of membrane lipid homeostasis is highly important in AD because amyloid precursor protein is a transmembrane protein, and membrane lipid dysregulation may affect the processing of this protein, leading to increased Aβ production [46]. OSA has been shown to increase oxidative stress [35]. Elevated lipid peroxidation is evidenced in both the brain and blood of AD patients [17, 18]. Therefore, OSA, by increasing lipid peroxidation, may disrupt membrane lipid homeostasis and contribute to AD pathogenesis. Supporting this notion, in our study, we found that several significantly altered lipid species were oxidized, which may have been caused by increased lipid peroxidation due to a more severe OSA. In addition, dysregulation regarding PL plasmalogens was also associated with the severity of OSA in AD. PL plasmalogens are a class of lipids enriched in cellular membranes that have been suggested to protect other membrane lipids against lipid peroxidation. Furthermore, our data also linked altered plasma levels of a cardiolipin (CL (78:2)) to OSA severity. CL is a mitochondria-exclusive PL that is essential for mitochondrial functionality. Mitochondria are the main producers of reactive oxygen species (ROS), and disruption of their functionality, which is evidenced in AD, has strongly been related to an increase in ROS [47]. Additionally, membrane PLs are a major source of lipid mediators that have a fundamental role in biological processes such as inflammation. Therefore, PL dyshomeostasis may also contribute to elevated systemic inflammation, which is characteristic of both AD and OSA [48, 49]. Our results also linked several TG species to OSA severity in AD patients. TGs are neutral lipids reserved in intracellular organelles named lipid droplets (LDs). At the systemic level, LDs are mainly produced in adipose tissue, but all cells can produce these organelles. In the periphery, LDs not only serve as a lipid storage and supply but also affect physiological processes, such as inflammation, cell signalling and redox homeostasis. For example, LDs in various immune cell types contain a large amount of arachidonic acid, which can serve as a precursor for eicosanoid synthesis [50]. Interestingly, our data also associated an eicosanoid (Cis-8,11,14,17-ETA) with the severity of OSA in AD, highlighting the possible role of OSA-derived systemic inflammation as a contributor to AD pathogenesis.

The present study has some limitations. Due to the incipient state of this field of research, an exploratory approach was carried out with the objective of obtaining new knowledge from which new hypotheses can be generated. Therefore, a targeted methodology is needed to determine the validity of our findings in an independent sample of individuals, especially in those with severe OSA. We included patients with MMSE scores > 20, so the results should be extrapolated cautiously for patients in more advanced stages of the disease. The main strength of this study was the use of PSG, which is the gold standard test, as the diagnostic method for OSA and allowed the performance of correlation analysis with different PSG variables related to the severity of OSA. Another strong point is that it included a relatively large sample of subjects who were recruited consecutively, which contributes to the generalization of the data. The patients were evaluated according to clinical criteria and specific CSF biomarkers of AD, so we can ensure that the patients had both clinically and biologically evident AD.

## Conclusions

In this study, using undirected lipidomics, we identified a lipid profile in the plasma of severe OSA patients with mild–moderate AD that was correlated with different polysomnographic measures of OSA severity. PLs and GLs were the most dysregulated lipids concerning OSA severity in AD patients. The presence of several oxidized lipid species suggests that OSA may contribute to AD pathogenesis by increasing lipid peroxidation. In addition, we identified a lipidomic signature that allows the identification of subjects with AD and severe OSA in a population of AD with better accuracy than the STOP-Bang screening scale, which suggests that they could be used as plasma biomarkers for the management of OSA or for screening those patients who could subsequently benefit from undergoing a PSG in the face of a high suspicion of severe OSA.

## Supplementary Information

**Additional file 1: Table S1.** Lipid features of the differentially expressed unknown lipids between non-OSA and OSA patients. **Figure S1.** Boxplot with lipids with differential expression between non-OSA and OSA patients.

## Data Availability

The data reported in this manuscript are available within the article and/or its supplementary data. Additional data from NCT02814045 will be shared by request from any qualified investigator.
