# Supplementary material for: Blood-based lipidomic signature of severe obstructive sleep apnoea in Alzheimer’s disease
Source: Alzheimers Res Ther. 2022 Nov 3;14:163. doi: 10.1186/s13195-022-01102-8 (PMC9632042; doi:10.1186/s13195-022-01102-8)

## Blood-based lipidomic signature of severe obstructive sleep apnoea in Alzheimer's disease

Farida Dakterzada^1^, Iván D. Benítez^2,3^, Adriano Targa^2,3^, Anna Carnes^1^, Montse Pujol^2^, Mariona Jové^4^, Olga Mínguez^2^, Rafi Vaca^2^, Manuel Sánchez-de-la-Torre^5^, Ferran Barbé^2,3^, Reinald Pamplona^4^, Gerard Piñol-Ripoll^1*^

^1^ Unitat Trastorns Cognitius, Clinical Neuroscience Research, IRBLleida-Hospital Universitari Santa Maria Lleida (Spain).

^2^ Group of Translational Research in Respiratory Medicine, Hospital Universitari Arnau de Vilanova and Santa Maria, IRBLleida, Lleida, Spain.

^3^ Centro de Investigación Biomédica en Red de Enfermedades Respiratorias (CIBERES), Madrid, Spain.

^4^ Department of Experimental Medicine, University of Lleida-Lleida Biomedical Research Institute of Lleida (UdL-IRBLleida), E25198 Lleida, Spain.

^5^ Group of Precision Medicine in Chronic Diseases, University Hospital Arnau de Vilanova and Santa María, IRBLleida, Department of Nursing and Physiotherapy, Faculty of Nursing and Physiotherapy, University of Lleida, Lleida, Spain

^*^Corresponding author:

Gerard Piñol Ripoll

Cognitive Disorders Unit

Hospital Universitari Santa Maria.

Rovira Roure nº 44. 25198. Lleida. Spain

Telephone: 34-937-727222. Ext. 173. Fax: 34-976-727366

E-mail: gerard_437302@hotmail.com

**Table S1. Lipid features of the differentially expressed unknown lipids between non-OSA and OSA patients.**

Data are adjusted for age, sex and BMI. Lipids with FC <0.75 (downregulated) or >1.25 (upregulated) and p value <0.05 were considered. Features are represented as exact mass@retention time. Definition of abbreviations: BMI = body mass index; FC = fold change; RT = retention time; PL= phospholipid; GL= glycerolipid; SP= sphingolipid; FA= fatty acid.

**Figure S1. Boxplot with lipids with differential expression between non-OSA and OSA patients.**


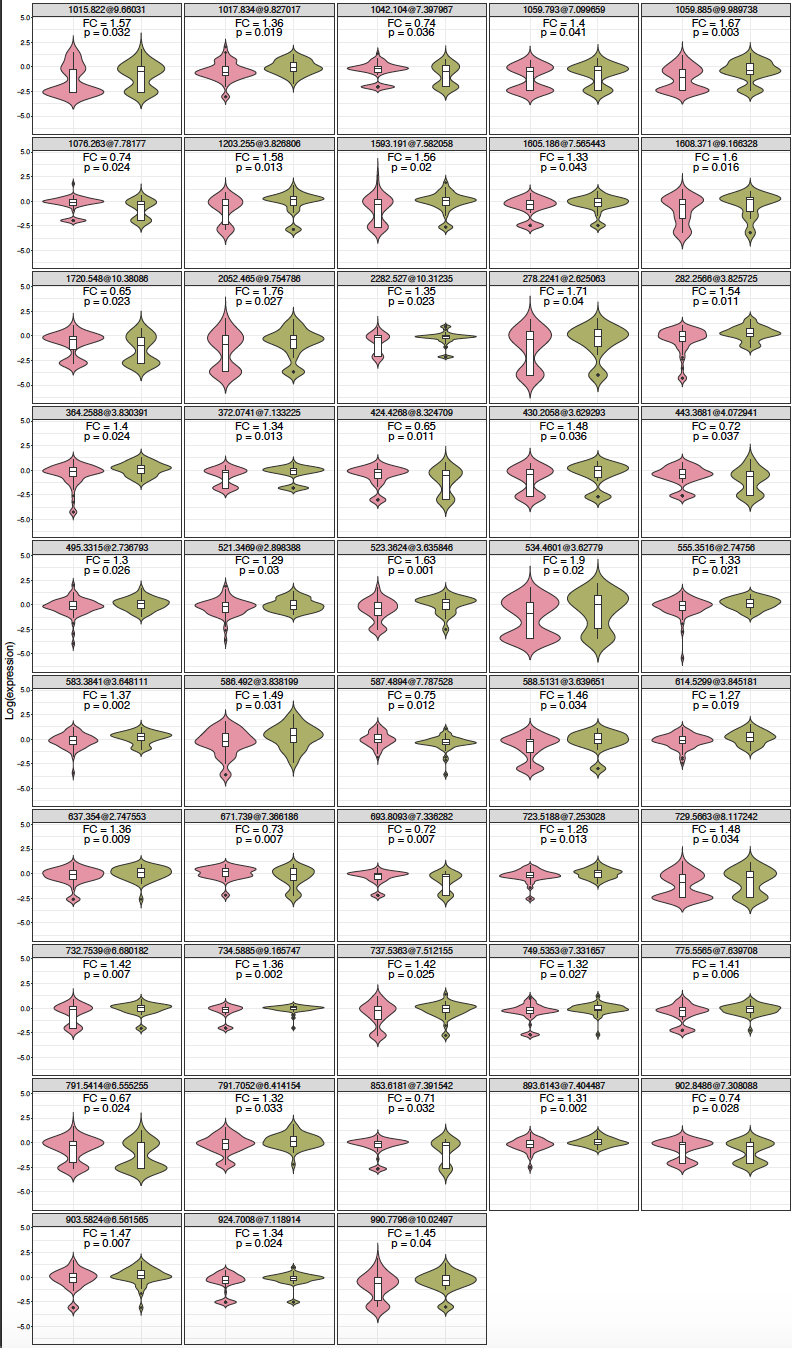

Supplement: Supplementary file 1 — Additional file 1: Table S1. Lipid features of the differentially expressed unknown lipids between non-OSA and OSA patients. Figure S1. Boxplot with lipids with differential expression between non-OSA and OSA patients. [file 13195_2022_1102_MOESM1_ESM.docx]
